# Bearing Capacity and Mechanical Behavior of an Innovative Bamboo Fiber-Reinforced Polymer Tendon with U-Head

**DOI:** 10.3390/polym13223976

**Published:** 2021-11-18

**Authors:** Xiang Li, Shuaiyu Zhao, Xinmiao Meng, Xiaodong Ji

**Affiliations:** Department of Civil Engineering, Beijing Forestry University, Beijing 100083, China; leexiang@bjfu.edu.cn (X.L.); Zsy201303101@bjfu.edu.cn (S.Z.)

**Keywords:** BFRP tendon, thin-shell model, thick-shell model, tension capacity, mechanical behavior

## Abstract

The development of steel in engineering structures or components faces the problems of high cost and high carbon emission, which demands new materials used as reinforcement to be proposed and applied. Bamboo is a green and renewable natural material, and has higher tensile strength parallel to grain compared to wood. Based on the excellent mechanical properties in the parallel-to-grain direction of bamboo fiber, this paper proposed an innovative bamboo fiber-reinforced polymer tendon with U-head (BFRP tendon). To analyze the tension capacity and mechanical behavior of the BFRP tendon, the thin-shell model and thick-shell model have been proposed in succession. Both models were compared with the results of tensile test and good agreement was achieved in the stress distribution and the ultimate load. The tension capacity of the BFRP tendon has been proved to be reliable, which can give full play to the advantages of bamboo fiber.

## 1. Introduction

**Figure 1:**
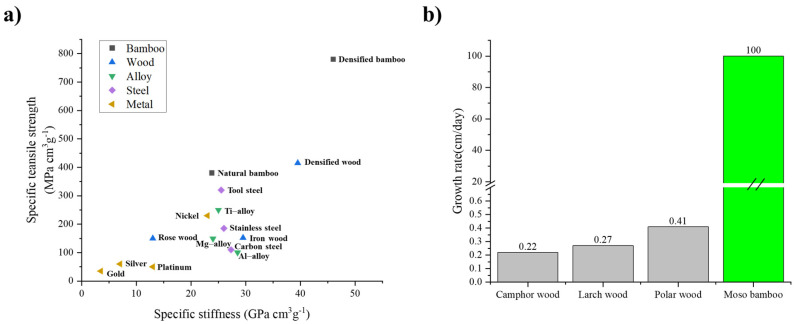
(**a**) Specific tensile strength versus specific stiffness of the densified bamboo compared to other natural materials, engineered steel, and strong metallic alloys [3]; (**b**) growth rates of typical bamboo and various wood species [3].

Steel applied in concrete constructions has risen exponentially due to its high strength, good durability and easy handling. However, the vast amount of steel used not only brings about economic cost problems, but also induces environmental problems such as carbon dioxide emissions during the manufacturing process. Developing new materials is one way to solve this problem. In recent years, the development of fiber-reinforced polymer (FRP) materials, such as carbon fiber-reinforced polymer (CFRP) and glass fiber-reinforced polymer (GFRP), offers great potential for application in concrete constructions [1,2]. Bamboo is a kind of natural FRP material, which possess strong carbon sequestration, low energy consumption, short growth cycle, high specific strength, and other excellent properties [3,4], as shown in Figure 1. In terms of the tensile strength parallel to grain that dominates the strengths of the reinforcement, some species of bamboo can reach 400 MPa, which is 1.7 times that of plain carbon steel [5]. In addition, the raw bamboo is very cheap. On account of the above characteristics of bamboo, it has been used in civil engineering to enhance the strength of concrete members and structures, such as beams and columns [6,7], slabs and walls [8,9], beam-column joints [10,11], and even the whole construction [12]. However, in most cases, bamboo as a reinforcing material in concrete structures is conservatively designed and the strength of bamboo fiber cannot be fully exploited.

The application of bamboo as a reinforcing material to concrete structures still faces three main problems. First, due to insufficient waterproofing measures or the difference in thermal expansion of materials, swelling and shrinkage of bamboo in concrete induce the reduction of bonding strength. Second, raw bamboo and its simple extensions such as bamboo strips are susceptible to transverse loading because of the absence of radial fibers. The last is that the bamboo tendons tend to be damaged in the concrete by termite and fungal [13]. Several measures have been conducted to improve the mechanical properties of bamboo-reinforced concrete, including the aspects of bonding strength, shear strength, flexural strength, water absorption, and durability [14,15].

Javadian et al. [5] studied about the bonding properties of a newly developed bamboo-composite reinforcement in concrete through pull-out test. Various coatings are applied to determine bonding behavior between concrete and the bamboo-composite reinforcement. It was found that bamboo-composite reinforcement without coating could provide enough bonding strength, but an epoxy coating with sand particles could provide extra protection without the loss of bonding strength. Mali et al. [16] studied the bonding strength of the bamboo-concrete interface under different surface treatments. It was concluded that the bamboo treated with different chemical coatings, sandblasting, and steel wire wrappings, could achieve the bonding strength 6–16 times than the untreated one. Ghavami [15] investigated bonding strength of treated and untreated raw bamboo splits in concrete subjected to pull-out test. It was indicated that a two-component epoxy resin coating could enhance the bonding strength 5.29 times, compared with that of untreated segments.

Mali et al. [7] discussed the flexural behavior of concrete beam with different reinforcement measures according to the results of four-point bending test. It was observed that concrete beams with 2.8% or with 3.8% longitudinal bamboo reinforcement had shown significantly higher shear and flexural strength than plain cement concrete beams. However, the beams with 2.8% bamboo reinforcement had less shear and flexural capacity compared to that of steel-reinforced concrete beams. Umeonyiagu et al. [17] predicted the flexural strength, tensile strength and the costs of bamboo-reinforced concrete material which have various curing days and percentage bamboo content using artificial neural network (ANN) and non-dominated sorting genetic algorithm-II (NSGA-II). As a result, the ANN model predicted the experimentally determined values of the tensile strength, flexural strength and the costs of bamboo-reinforced concrete material excellently with correlation coefficients of 0.99635, 0.99739, and 1, respectively. The prediction model was then optimized through NSGA-II combined with the fitness function (i.e., ANN).

Hu [18] studied the effects of ten different water-repellent coating methods used for bamboo material in bamboo-reinforced concrete through water absorption test. Three coating methods for bamboo, rosin glyceride-bitumen mixture painted, rosin glyceride and bitumen painted in turn, as well as rosin glyceride and white lead paint (8% white lead) painted in turn, have been recommended. In addition, the first one is the most economical, the last one is the most waterproofing. Kute et al. [19] studied the reduction of water absorption of bamboo without affecting the bonding strength of bamboo-reinforced concrete at a low cost. Oil paint, bitumen kerosene paint, and readymade bituminous paint, were applied to form an impervious layer over the surface of bamboo. Through water absorption test, the conclusions showed that the readymade bituminous paint was most effective for countering water absorption, in which the reduction was 75%.

Fang et al. [20] analyzed the influence of heat treatment on the chemical transformation and the durability of bamboo fiber and its reinforced composites through chemical component analysis and UV absorption analysis. The result showed that heat treatment was helpful to improve the durability of bamboo fiber due to the decrement of the equilibrium moisture content. Lima et al. [21] investigated the durability of bamboo using as concrete reinforcement by evaluating the tensile strength and Young’s Modulus of bamboo under various cycles. From the result, neither the bamboo tensile strength nor the Young’s Modulus of bamboo decreased under 60 cycles of wetting and drying in solution of calcium hydroxide and tap water. Besides, the averaged tensile strength of bamboo is approximately 280 MPa in the specimens without node and 100 MPa in the specimens with node. Ghavami et al. [15] tested a steel-reinforced concrete column after 10 service years with a bamboo-reinforced concrete beam after 15 service years. It was observed that the section of the bamboo-reinforced beam was still in a satisfactory state after 15 years due to the mothproofing and bonding treatment. The tensile strength of the bamboo in the concrete beam was only slightly degraded compared to the original bamboo. However, the steel bars in the column were severely corroded. From the above researches, it can be seen that the defects of bamboo as a reinforcing material in concrete have been overcome to a certain extent. Nonetheless, it is still difficult to find a particular anchoring system that can utilize the full tensile strength of bamboo.

In this study, a new bamboo FRP tendon (BFRP tendon) with U-head is proposed to be applied in the concrete members and constructions. Compared with the existing bamboo tendons, the bearing capacity of the BFRP tendon is not only provided by the bonding strength of the bamboo-concrete interface, but also from the U-shaped heads at the anchor ends. This structural design ensures the utilization of the tensile strength parallel to grain of bamboo fiber and avoids the defects on the rod due to the lateral pressure. Besides, it has already been used in other FRP tendons. Djamaluddin et al. [22] presented an innovative CFRP rods with U-anchor for concrete structures, and researched the mechanical behavior through tensile test. The results indicated that the fracture occurred in the circular loop of U-anchor, and the capacity of U-anchor was approximately 65% of the rod tensile strength. Fan et al. [23] presented a CFRP single-strap ground anchor, and studied its capacity and load transfer mechanism through the pull-out tests. The results indicated that the ultimate load could reach to 529 kN, and the grout with a compression strength of about 60 MPa was destroyed at the same time.

To figure out the tension bearing capacity and mechanical behavior of the BFRP tendon, theoretical analysis and tensile tests were conducted in this research. The BFRP tendon were manufactured by winding bamboo strips and wrapping with GFRP sheet. The GFRP coating would improve the durability and bonding strength of the inner BFRP rods.

## 2. Materials and Manufacturing Method

The raw materials used in the manufacturing of BFRP tendons include moso bamboo (*Phyllostachys Pubescens*) strips, ethyl cyanoacrylate adhesive, epoxy resin adhesive, and glass fiber sheet. The moso bamboo strips were all 3 to 4 years old and were supplied by the manufacturer in Lantian, Fujian, China. The ethyl cyanoacrylate adhesive was supplied by Guangdong Heyi Glue Co., Ltd, China. The epoxy resin adhesive were supplied by Shanghai Autun chemical technology Co., Ltd, China, and the glass fiber sheet were supplied by Nanjing Hitech Composites Co., Ltd, China. The strength parallel to grain of moso bamboo is general ranging from 100 to 300 MPa in accordance with the location from the inner to the outer wall of the bamboo culm, and its elastic modulus is about 11 GPa. However, the strength of bamboo with nodes decreases to 100–200 MPa [24,25,26]. The mechanical properties of the other materials are listed in Table 1, which were provided by material suppliers.

**Figure 2:**
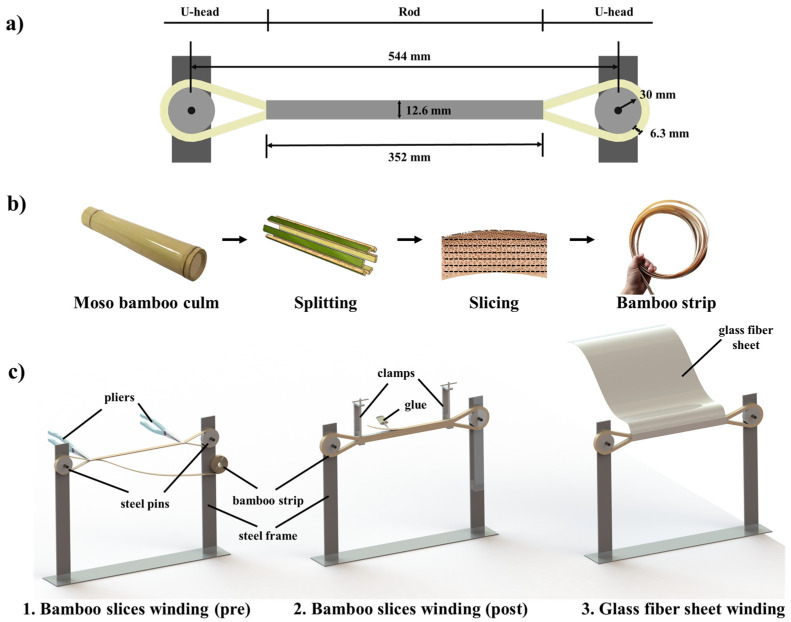
(**a**) Dimension of the BFRP tendon specimens; (**b**) manufacturing process of the bamboo strips; (**c**) manufacturing process of the BFRP tendon specimens.

The BFRP tendon specimens were made by turning bamboo strips longitudinally on two steel pins with a diameter of 60 mm. The U-head of the BFRP tendon formed at the two pins 544 mm apart. Meanwhile, a rod with a length of 352 mm formed. The width of the bamboo strips used for manufacturing was 15 mm and the thickness was 0.7 mm. After nine turns of bamboo strips, the thickness of the U-head was about 6.3 mm, and the rod without GFRP sheet wrapping was about 12.6 mm in thickness. The dimensions and the manufacturing process of the BFRP tendon specimens are shown in Figure 2.

The last procedure is the wrapping of the GFRP sheet. In this process, the rod is wrapped three times by glass fiber sheet mixed with epoxy resin to prevent the BFRP tendon from disassembly due to unfavorable load conditions. This phenomenon might occur at the place where the bamboo strip was separated at the first turn. Moreover, the wrapping of GFRP sheet has more pronounced effect on the BFRP tendon in concrete, which is beneficial to the bonding strength and durability. The GFRP sheet can be wrapped around the entire bamboo tendon, including the U-head, to facilitate reinforcement in future work.

## 3. Theoretical Analysis

### 3.1. The Thin-Shell Model

**Figure 3:**
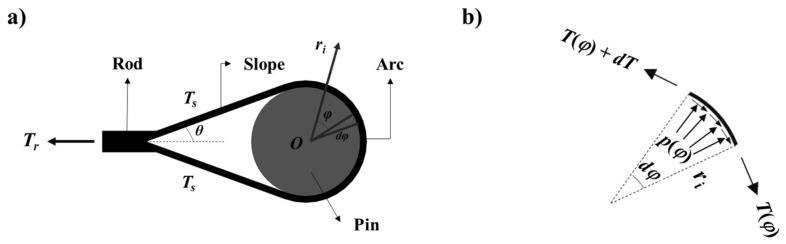
(**a**) Mechanical model of the U-head; (**b**) stress state of a micro-element of the arc.

The mechanical behavior of the BFRP tendon mainly occurs in the rod and the U-head. This chapter will analyze the two structures based on the theory of elasticity. During the tensile test, the rod is in uniaxial tension, and a linear model can explain its stress–strain constitutive relationship with a constant elastic modulus. For the U-head composed of a slope and an arc, its mechanical model is shown in Figure 3a. Assuming that *T_r_* is the tension of the rod and *θ* is the angle between the rod and the slope. Due to the thickness of the U-head being very small compared to the diameter of the pin, the U-head structure can be simplified to a thin-shell model (i.e., the stress distribution is uniform in the cross-section).

According to the equilibrium, the tension of each branch (*T_s_*) can be obtained:

(1) $$T_{s}=\frac{T_{r}}{2\;cos\theta }$$

A cylindrical coordinate system has been established by taking the center of the arc as the origin and the plane of the starting point of the arc–pin interface as the coordinate axis of *φ* = 0 (Figure 3a). Based on this, a micro-element with its stress state has been taken out from the arc, as shown in Figure 3b. Both the circumferential tension (*T*) and the radial pressure (*p*) of the arc can be expressed as position function related to the angle (*φ*). Assuming that there is a friction existed on the arc–pin interface with a constant friction coefficient, the equilibrium equation of the circumferential and the radial directions can be obtained:

(2) $$dT=-\mu p\left(\varphi \right)r_{i}d\varphi$$

(3) $$\left[T\left(\varphi \right)+T\left(\varphi \right)+dT\right]sin\frac{d\varphi }{2}=p\left(\varphi \right)r_{i}d\varphi$$

Here, *r_i_* is the inner semidiameter of the arc and *μ* is the friction coefficient. Combining the above three equations and solving the differential equation after subtracting the high-order infinitesimal, the formula for the tension distribution along the arc can be obtained:

(4) $$T\left(\varphi \right)=T_{s}e^{-\mu \varphi }$$

The radial pressure can be derived using Equations (2) and (4), as follows:

(5) $$p\left(\varphi \right)=\frac{T_{s}e^{-\mu \varphi }}{r_{i}}$$

**Figure 4:**
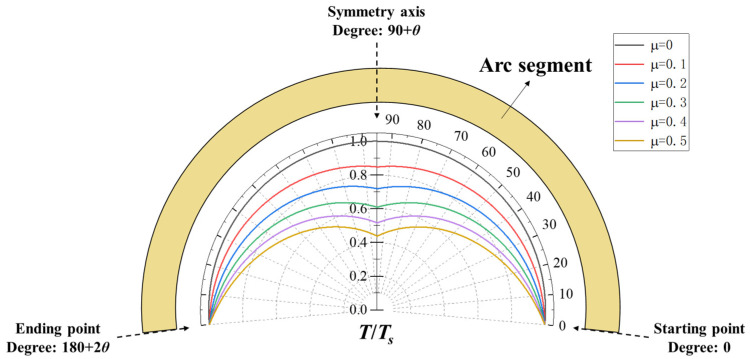
Tension distribution of the arc under different friction coefficients.

Equations (4) and (5) are the Euler-Eytelwein equations of the pulley model, which were developed to describe friction of a rope or flat belt surrounding a cylindrical drum. Considering the symmetry, the tension distribution along the arc under different friction coefficients can be obtained according to Equation (4), as shown in Figure 4.

**Figure 5:**
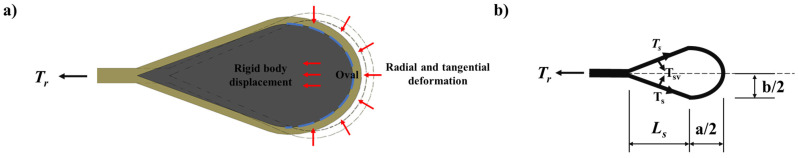
(**a**) Deformation and displacement of the U-head and the pin under tension; (**b**) deformation-considered mechanical model of the U-head.

As Figure 4 shows, when the arc–pin interface is frictionless, the tension of the arc is a constant value (i.e., the tension of the branch (*T_s_*)). However, when the interface is rough, the tension gradually decreases along the arc from the starting point (*φ* = 0 and *φ* = 180 + 2*θ*) to the apex (*φ* = 90 + *θ*) where possesses the minimum, and the rate of tension decline increases as the friction coefficient increases. It can be further seen that the influence of the friction coefficient on the rate of tension decline weakens with the increase of the friction coefficient, and it can be proved mathematically that this influence will gradually converge to zero. For the *p*(*φ*), the value of it is equal to 1/*r* times the tension at the same point. Therefore, the radial pressure distribution is the same as the tension distribution shown in Figure 4. Moreover, if the elastic deformation is considered, the pin will deform inwardly under the action of radial pressure to form an ellipse, as shown in Figure 5a. Correspondingly, the mechanical model of the U-head is changed as shown in Figure 5b.

We assume that *φ* = 0 coincides with the minor axis of the ellipse. Based on the equilibrium of tension in the orthogonal directions, the distribution of the tension and the radial pressure can also be obtained:

(6) $$T\left(\varphi \right)=T_{s}e^{-\mu \varphi }$$

(7) $$p\left(\varphi \right)=\frac{T\left(\varphi \right)}{r\left(\varphi \right)}=\frac{abT_{s}e^{-\mu \varphi }}{{\left(a^{2}{\;cos}^{2}\varphi +b^{2}{sin}^{2}\varphi \right)}^{\frac{3}{2}}}$$

where *r*(*φ*) is the function of the radius of curvature of the ellipse with respect to the angle, and *a* and *b* are the lengths of the major and minor axes of the ellipse. It can be seen from Equations (6) and (7) that when the radial pressure causes the pin to deform into an ellipse, the tension of the arc does not change. However, due to the change of the radius of curvature, the radial pressure is no longer a constant. Along the fiber direction, the radius of curvature at the starting point becomes larger, and the radial pressure becomes smaller, correspondingly. At the apex of the arc, the reverse applies.

We assume that the deformation of the steel pin under the ultimate load of the BFRP tendon is negligible in the tensile test. Therefore, combined with the area relationship of each section and Equations (1) and (6), the stress distributions of the rod (*σ_r_*), the slope (*σ_s_*) and the arc (*σ_a_*), as well as the ultimate load of the BFRP tendon (*P*), can be obtained respectively:

(8) $$\sigma_{r}=\frac{T_{r}}{2A};\sigma_{s}=\frac{T_{r}}{2\;cos\theta A};\sigma_{a}=\frac{T_{r}e^{-\mu \varphi }}{2\;cos\theta A}$$

(9) $$P=2tw\;cos\theta S_{t}$$

where *A* represents the cross-sectional area of the U-head, and its value is half of the rod; *t* and *w* represent the thickness and width of the BFRP tendon respectively; *S_t_* represents the strength parallel to grain of the bamboo strip. It is obvious that *σ_s_* is the maximum among them, which means the critical section of the BFRP tendon is in the slope in theory. Moreover, based on the assumption of the elastic body, the strain distribution of the U-head is consistent with the stress distribution.

Herein, it can be concluded that when subjected to unidirectional tension, the weakest link arises in the slope. Moreover, the circumferential tension of the arc decreases from the starting point to the apex according to Equation (4) or (6) (range of degree: 0–90 + *θ*). At the same time, the radial pressure of the arc varies depending on whether deformation is considered or not. When considering deformation, the radial pressure cahnges linearly with the tension. On the contrary, it is controlled by the tension and the curvature.

### 3.2. The Thick-Shell Model

**Figure 6:**
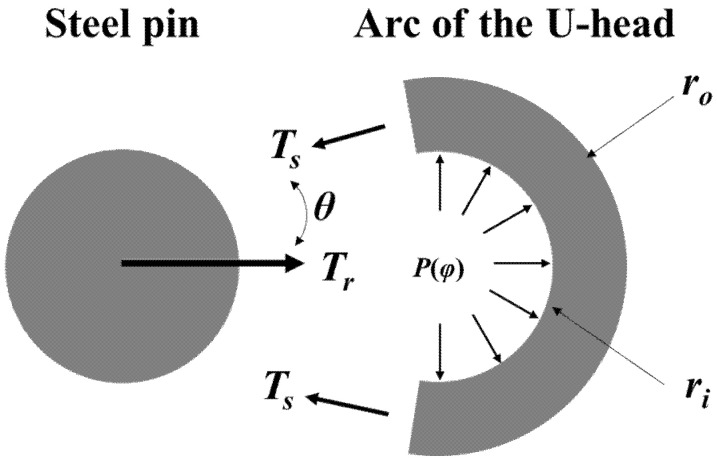
The thick-shell model.

The thin-shell model in Section 3.1 cannot analyze the stress distribution along the thickness direction of the arc, so the thick-shell model is needed to be introduced. Regardless of friction and deformation, the radial pressure is a constant value according to Equation (5). The thick-shell model is shown in Figure 6. Since the stress distribution is independent of the circumferential position of the micro-element, it is an axisymmetric plane problem.

The stress function is simplified as a univariate function (i.e., *Ψ* = *Ψ*(*r*)). The stress function *Ψ* derived from the equilibrium equation in the cylindrical coordinate system is as follows:

(10) $$\sigma_{r}=\frac{1}{r}\frac{\partial \Psi }{\partial r}+\frac{1}{r^{2}}\frac{\partial^{2}\Psi }{\partial \theta^{2}}\;\sigma_{\theta }=\frac{\partial^{2}\Psi }{\partial r^{2}}\;\tau_{r\theta }=-\frac{\partial^{2}}{\partial r\partial \theta }\left(\frac{\Psi }{r}\right)$$

*σ_r_*, *σ_θ_*, and *τ_rθ_* present the radial stress, the circumferential stress, and the shear stress, respectively. Based on this, the stress function can be rewritten:

(11) $$\sigma_{r}=\frac{1}{r}\frac{\partial \Psi }{\partial r}\;\sigma_{\theta }=\frac{\partial^{2}\Psi }{\partial r^{2}}\;\tau_{r\theta }=0$$

For the orthotropic plane problem, the constitutive relation is as follows:

(12) $$\left[\begin{array}{c} \varepsilon_{r}\\ \varepsilon_{\theta }\\ \varepsilon_{r\theta } \end{array}\right]=\left[\begin{array}{ccc} \frac{1}{E_{r}} & -\frac{\nu_{\theta r}}{E_{\theta }} & 0\\ -\frac{\nu_{r\theta }}{E_{r}} & \frac{1}{E_{\theta }} & 0\\ 0 & 0 & \frac{1}{G_{r\theta }} \end{array}\right]\cdot \left[\begin{array}{c} \sigma_{r}\\ \sigma_{\theta }\\ \sigma_{r\theta } \end{array}\right]$$

*E*, *G* is the elasticity and shear moduli, and *r*, *θ* indicate the radial and circumferential directions. Considering no volume force, the stress function should meet the compatibility equation as follows:

(13) $$\left(\frac{\partial^{2}}{\partial r^{2}}+\frac{1}{r}\frac{\partial }{\partial r}+\frac{1}{r^{2}}\frac{\partial }{\partial \theta^{2}}\right)\left(\frac{\partial^{2}}{\partial r^{2}}+\frac{1}{r}\frac{\partial }{\partial r}+\frac{1}{r^{2}}\frac{\partial }{\partial \theta^{2}}\right)\Psi =0$$

According to Equations (11)–(13), the compatibility equation expressed by stress function can be obtained:

(14) $$\left(\frac{\partial^{4}}{\partial r^{4}}+\frac{2}{r}\frac{\partial^{3}}{\partial r^{3}}-k^{2}\frac{1}{r^{2}}\frac{\partial^{2}}{\partial r^{2}}+k^{2}\frac{1}{r^{3}}\frac{\partial }{\partial r}\right)\Psi =0$$

In Equation (14),

(15) $$k=\sqrt{\frac{E_{\theta }}{E_{r}}}$$

The general solution of the stress function can be obtained:

(16) $$\Psi =Ar^{1+k}+Br^{1-k}$$

Combining with the boundary conditions,

(17) $${\left[\sigma_{r}\right]}_{r=r_{o}}=0,\;{\left[\sigma_{r}\right]}_{r=r_{i}}=-p_{i}$$

The coefficients *A* and *B* can be obtained:

(18) $$A=\frac{{\left(\frac{r_{i}}{r_{o}}\right)}^{k+1}p_{i}}{\left(1+k\right)r_{o}{}^{k-1}\left[1-{\left(\frac{r_{i}}{r_{o}}\right)}^{2k}\right]}\;B=\frac{p_{i}}{\left(1-k\right)r_{i}{}^{-k-1}\left[{\left(\frac{r_{i}}{r_{o}}\right)}^{2k}-1\right]}$$

According to Equations (11), (16) and (18), the expressions of radial stress and circumferential stress can be obtained:

(19) $$\begin{array}{c} \sigma_{r}\left(r\right)=\frac{{\left(\frac{r_{i}}{r_{o}}\right)}^{k+1}p_{i}}{1-{\left(\frac{r_{i}}{r_{o}}\right)}^{2k}}{\left(\frac{r}{r_{o}}\right)}^{k-1}-\frac{p_{i}}{1-{\left(\frac{r_{i}}{r_{o}}\right)}^{2k}}{\left(\frac{r_{i}}{r_{o}}\right)}^{k+1}{\left(\frac{r_{o}}{r}\right)}^{k+1}\\ \sigma_{\theta }\left(r\right)=\frac{{\left(\frac{r_{i}}{r_{o}}\right)}^{k+1}p_{i}}{1-{\left(\frac{r_{i}}{r_{o}}\right)}^{2k}}k{\left(\frac{r}{r_{o}}\right)}^{k-1}+\frac{p_{i}}{1-{\left(\frac{r_{i}}{r_{o}}\right)}^{2k}}k{\left(\frac{r_{i}}{r_{o}}\right)}^{k+1}{\left(\frac{r_{o}}{r}\right)}^{k+1} \end{array}$$

When *k* = 1, the material is isotropic. According to the equilibrium in the horizontal direction, the tension of the rod (*T_r_*) can be obtained:

(20) $$T_{r}=\int_{-\left(90+\theta \right)}^{90+\theta }p_{i}r_{i}\;cos\varphi d\varphi =2p_{i}r_{i}\;cos\theta$$

By substituting Equation (20) into (19), the equation between the tension of the rod and the radial and circumferential stresses of the arc can be obtained:

(21) $$\sigma_{r}\left(r\right)=\frac{r_{i}^{k+1}\left(r^{2k}-r_{o}^{2k}\right)}{r^{k+1}\left(r_{o}^{2k}-r_{i}^{2k}\right)}\frac{T_{r}}{2r_{i}\;cos\theta }\;\sigma_{\theta }\left(r\right)=\frac{r_{i}^{k+1}\left(r^{2k}+r_{o}^{2k}\right)}{r^{k+1}\left(r_{o}^{2k}-r_{i}^{2k}\right)}\frac{T_{r}}{2r_{i}\;cos\theta }$$

According to Equation (21), when the uniformly distributed pressure *p_i_* acts on the inner surface of the arc, all positions of the arc is in tension, and the innermost (*r* = *r_i_*) one has the most extensive circumferential stress. Meanwhile, the radial stress is compressive at all positions of the arc, and the innermost one reaches maximum. The circumferential and radial stresses both decrease gradually from the inner to the outer along the thickness direction. The maximum (*σ_θ_*_,max_(*r*)) and minimum (*σ_θ_*_,min_(*r*)) values of circumferential stress can be obtained:

(22) $$\sigma_{\theta ,max}\left(r\right)=\sigma_{\theta }\left(r_{i}\right)=\frac{\left(r_{i}^{2k}+r_{o}^{2k}\right)}{\left(r_{o}^{2k}-r_{i}^{2k}\right)}\frac{kT_{r}}{2r_{i}\;cos\theta }\;\sigma_{\theta ,min}\left(r\right)=\sigma_{\theta }\left(r_{o}\right)=\frac{r_{i}^{k}r_{o}^{k}}{\left(r_{o}^{2k}-r_{i}^{2k}\right)}\frac{kT_{r}}{r_{o}\;cos\theta }$$

The uniformity coefficient of circumferential stress distribution (*λ*) can be obtained:

(23) $$\lambda =\frac{\sigma_{\theta ,max}}{\sigma_{\theta ,min}}=\frac{\sigma_{\theta }\left(r_{i}\right)}{\sigma_{\theta }\left(r_{o}\right)}=\frac{1+{\left(\frac{r_{o}}{r_{i}}\right)}^{2k}}{2{\left(\frac{r_{o}}{r_{i}}\right)}^{k-1}}$$

**Figure 7:**
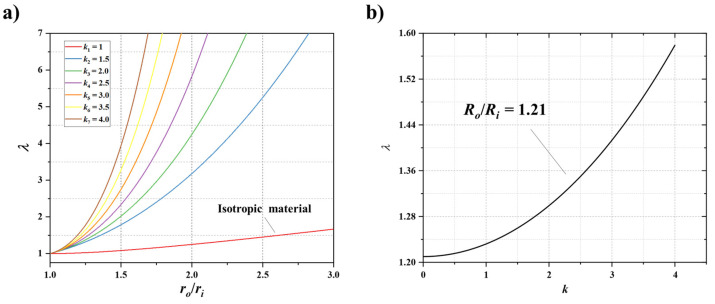
(**a**) Relationship between *λ* and *r_o_*/*r_i_* under different *k*; (**b**) relationship between *λ* and *k* when *r_o_*/*r_i_* = 1.21.

According to Equation (23), it can be seen that *λ* is related to the ratio of outer semidiameter to inner semidiameter (*r_o_/r_i_*) and the value of *k*. The relationship is shown in Figure 7a. *λ* increases as *r_o_/r_i_* or *k* increases. Under the same *r_o_/r_i_*, the greater the value of *k*, the higher the anisotropy. Moreover, reducing *r_o_/r_i_* helps to improve the uniformity of circumferential stress. In this research, the arc were fabricated with the *r_o_/r_i_* = 1.21. Based on it, the *λ*–*k* relationship curve can be obtained, as shown in Figure 7b. It can be seen that the variation of *λ* with the value of *k* is relatively small. For most anisotropic materials, it can be proved that the value of *k* is not greater than 4. Therefore, when the *r_o_/r_i_* = 1.21, the value of *λ* of bamboo is 1–1.58. According to Equation (22), the tension capacity of the BFRP tendon (*P*) can also be obtained:

(24) $$P=\frac{r_{o}^{2k}-r_{i}^{2k}}{r_{o}^{2k}+r_{i}^{2k}}\frac{2r_{i}w\;cos\theta }{k}S_{t}$$

### 3.3. Discussion on Two Theoretical Model

In the Section 3.2 and Section 3.3, the thin-shell model and thick-shell model for the arc were introduced to analyze the stress distribution and ultimate load of the BFRP tendon. The thick-shell model is more practical than the thin-shell one due to the consideration of the thickness. However, the friction was ignored in the former. Taking the dimension of the BFRP tendon specimens used in this research as an example, the tension capacity is calculated by the two theoretical models in this section. The thickness (*t*) and width (*w*) of the U-head are 6.3 and 15 mm, respectively; the inner semidiameter (*r*_i_) and outer semidiameter (*r*_o_) of the arc are 30 and 36.3 mm, respectively; the inclination angle (*θ*) of the slope is 17°. Moreover, we assume that the BFRP tendon is isotropic structure and the strength parallel to grain (*S*_t_) is 100 MPa.

The tension capacity of the BFRP tendon can be calculated:

(25) $$P_{1}=2tw\;cos\theta S_{t}=18.07\;\mathrm{kN}\;P_{2}=\frac{r_{o}^{2k}-r_{i}^{2k}}{r_{o}^{2k}+r_{i}^{2k}}\frac{2r_{i}w\;cos\theta }{k}S_{t}=16.21\;\mathrm{kN}$$

It can be seen that the theoretical solution based on the thick-shell model is relatively low. The reason is that the stress distribution of the thick-shell model is uneven along the thickness, and the initial fracture occurs in the innermost layer rather than the whole cross-section. The two theoretical solutions of tension capacity can also be used for later analysis and verification.

## 4. Tensile Test

### 4.1. Test Set-Up

**Figure 8:**
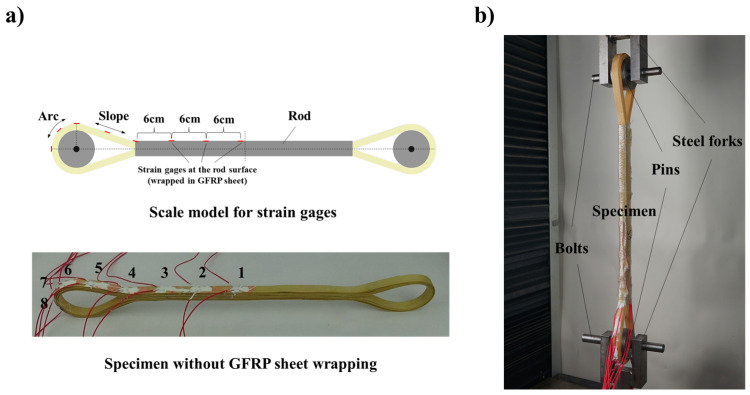
(**a**) Positions of eight strain gages on the surface of the BFRP tendon specimen; (**b**) the entire test set-up for tensile test.

According to the symmetry, eight strain gages were stuck to the surface of the BFRP tendon for verification, as shown in Figure 8a. The type of strain gage was BX120-5AA, produced by Zhejiang Huangyan Testing Instrument Factory, China. The resistance and the sensitivity coefficient of the gages were 119.9 ± 0.1 Ω and 2.08 ± 1%, respectively. Three strain gages were stuck to the rod. The other five strain gages were stuck to the U-head, which were located at the starting point of the slope, the ending point of the slope (i.e., starting point of the arc), the apex of the arc, and their two midpoints. In addition, the resistance wires of the strain gages were protected by silicone rubber with a relatively small elastic modulus.

The measure range of the universal testing machine used in the tensile test was 0–100 kN. To guarantee the BFRP tendon specimen born the load directly, the steel fork was designed and manufactured for the extension. The entire test set-up is shown in Figure 8b. According to the manufacturing method described in Section 2, nine BFRP tendon specimens were fabricated, and the relevant parameters are shown in Table 2. The BFRP tendon specimens can be divided into two batches depended on whether the bamboo strips used for manufacturing were close to the bamboo green of the raw moso bamboo. Based on it, we named the BFRP tendon specimens accordingly. For instance, the Specimen C1 means the bamboo strips used to fabricate the first specimen were relatively close to the bamboo green, and the “F” was marked with the opposite meaning. The load was applied with a rate of 2 mm/min until the final failure of the BFRP tendon occurred. It should be noted that Specimen C2 used ethyl cyanoacrylate adhesive instead of epoxy resin adhesive to wrap the GFRP sheet and the friction of the arc–pin interface of the Specimen A4 was increased by controlling the lubricant thickness.

### 4.2. Test Results

**Figure 9:**
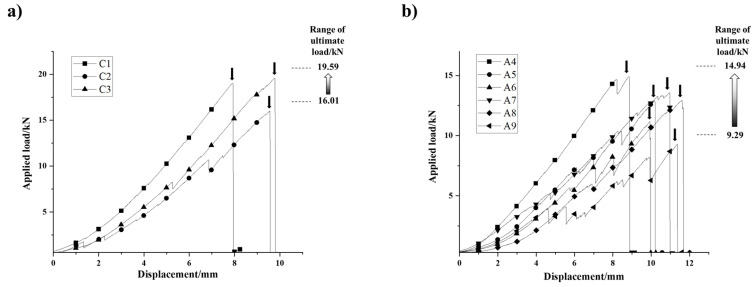
(**a**) Loading curves of three BFRP tendon specimens of batch C; (**b**) loading curves of six BFRP tendon specimens of batch A.

Figure 9a shows that the applied load and the displacement relationship of three BFRP tendons of batch C (batch Cs) during the tensile test. The ultimate loads and their displacements were recorded in Table 2. The failure of the batch Cs was brittle fracture, and the linear relationship between applied load and displacement before failure was significant. The averaged ultimate load of batch Cs was 18.22 kN, and Specimen C2 was relatively large than the other two specimens. However, there is no significant difference in the ultimate displacement of batch Cs.

The loading curves of six BFRP tendon specimens of batch A (batch As) during the tensile test were also be obtained, as shown in Figure 9b. Their mechanical behavior can be also described as linearity and brittleness. The averaged ultimate load of batch As was 12.5 kN. Among the batch As, Specimen A5 with greater friction was not significantly different in ultimate load and its displacement from others, and the localized fracture was observed in A6 and A9 before the final failure.

**Figure 10:**
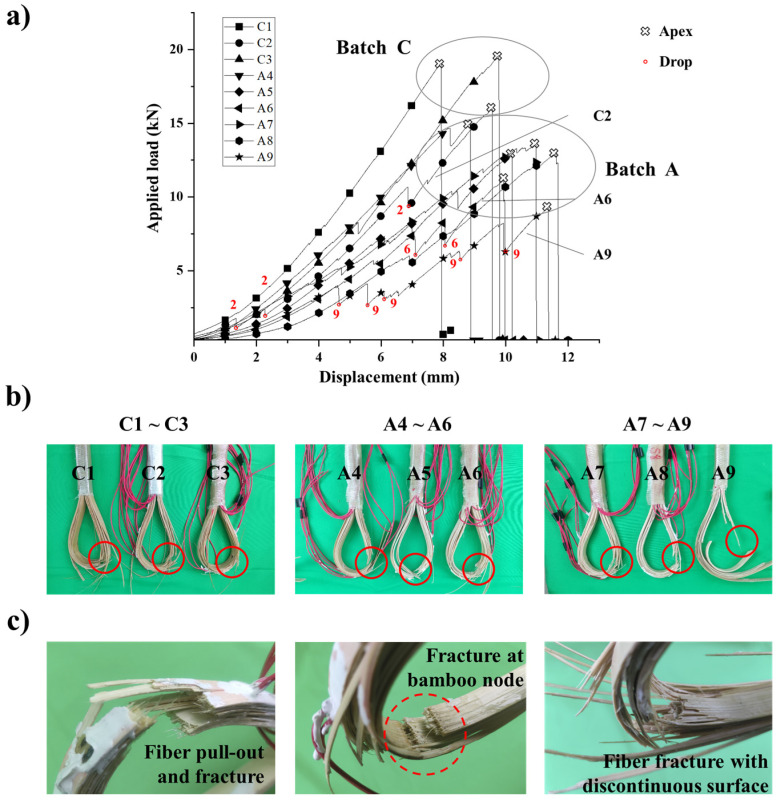
(**a**) Loading curves of nine BFRP tendon specimens with particular marks; (**b**) failure modes of nine BFRP tendon specimens; (**c**) details of the failure surfaces.

We putted the loading curves of nine BFRP tendon specimens together for further analysis (Figure 10). It is obvious that batch Cs and batch As have differences on both the ultimate load and corresponding displacement. The tension capacity of batch Cs (i.e., the ultimate load) are significantly higher than batch As. Except for the particular specimens shown in Table 2, the averaged tension capacity of batch Cs is 19.32 kN, which is 1.85 times that of batch A. Moreover, the loading curves of batch Cs are steeper than those of batch As, which indicates that batch Cs possessed relatively high elasticity. However, batch As were superior in ductility according to the averaged ultimate displacement which is 15% higher than batch Cs. During the tensile test, premature failure due to the fracture of outmost bamboo strip was observed in the Specimen C2, A6, and A9. This phenomenon can also be reflected on the loading curves. Each curve of Specimen C2, A6, and A9 shows several obvious sudden drops with the increase of displacement, which is usually accompanied with localized failure of structure (shown in Figure 10a). It is worth noting that the loading curve of Specimen A4 shows similar characteristic, and it failed shortly after the second drop.

### 4.3. Model Verification

**Figure 11:**
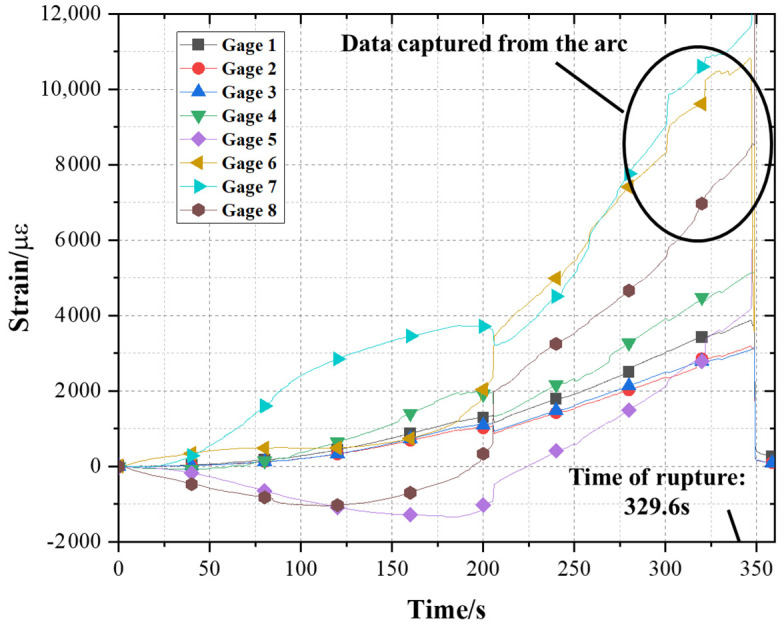
Strain curves of specimen A7 in eight positions of strain gages.

Figure 10b shows the failure modes of all specimens. It can be concluded that the failure of the BFRP tendon specimens occurred on the U-head in the tensile test, more specifically, near the junction of the arc and the slope. This phenomenon is completely consistent with the introduced two theoretical models. In addition, it could be found that the failure surfaces depended on the existence of bamboo nodes. Overall, the bamboo fibers were pulled out and fractured. The failure surface was rough and discontinuous. Nonetheless, when the bamboo node was located at the critical section, the failure surface was neat as shown in Figure 10c. The accuracy of the theoretical models can also be verified by the data of strain gages. Taking specimen A7 as an example, the strain data of eight points during the tensile test were captured, as shown in Figure 11.

According to the data of strain gages, the strains of the three points on the arc were greater than those of the slope and/or the rod, and the strain of the apex of the arc (Gage 8) was the lowest among the three points (i.e., 6, 7 and 8). It has been proved through the theoretical analysis. The data collected at each strain gage fluctuated with the increase of tensile loading, especially in the early stage, which was caused by relative slip after debonding between some bamboo strips.

The tension capacity of the BFRP tendon obtained from the tests can be compared with the two theoretical solutions (i.e., data in Table 2 compared with Equation (22)). The comparison result is shown in Table 3. The calculated tension capacity based on the thin-shell model and the thick-shell model are in good agreement with the test result of batch C, with the errors of 0.8% and 11%, respectively. However, the agreement between the models and the test result of batch A is not strong, with the errors of 44% and 30%, respectively. The reason is that the bamboo strips close to the bamboo green of raw moso bamboo have greater strength parallel to grain, which is more in line with the assumption of elastic modulus in the theoretical solutions of the two models.

## 5. Conclusions

Based on the research in this paper, the following conclusions can be drawn:

1. The thin-shell model has been verified to be accurate to analyze the tension capacity and the mechanical behavior of the BFRP tendon. The error between the tensile result and the theoretical solution of the thin-shell model is only 0.8%. Under the tensile loading, the maximum stress occurs in the slope. Moreover, when the friction is considered, the circumferential stress decreases gradually from the starting point of the arc–pin interface to the apex of the arc.
2. The thick-shell model has also been accepted to analyze the tension capacity and the mechanical behavior of the BFRP tendon. The error between the tensile result and the theoretical solution of the thick-shell model was 11%. Under the tensile loading, the circumferential stress of the arc is unevenly distributed along the thickness, more specific, it decreases from the innermost surface to the outermost one. The uniformity coefficient (*λ*) is determined by the thickness of the arc and the elastic modulus of bamboo strips. Moreover, when *r*_o_/*r*_i_ = 1.21, the *λ* of bamboo can be inferred between 1–1.58.
3. Based on the theoretical solutions and the result of tensile test, the weakest link of the BFRP tendon has been proved to be at the junction of arc and the slope of the U-head under the tensile loading, and the failure of the BFRP tendon is brittle fracture. Moreover, the averaged tension capacity of the BFRP tendons which were manufactured by stronger bamboo strips (i.e., the bamboo strips were close to the bamboo green skin of the raw moso bamboo) was about 1.85 times than those of not.
4. In this research, the tension capacity of the BFRP tendon can reach 18 kN. The BFRP tendon can fully utilize the advantages of bamboo fiber, and strength parallel to grain was about 100 MPa based on the assumption of isotropy.

## Figures and Tables

**Table 1:** Mechanical properties of the materials.

Material	Elasticity Modulus (GPa)	Tensile Strength (MPa)	Shear Strength (MPa)
Ethyl cyanoacrylate adhesive	-	26	19
Epoxy resin adhesive	2.9	60.1	17.5
glass fiber sheet	75	1150	-

**Table 2:** Summary of tensile test on nine specimens.

Specimen	Full Length (mm)	Rod Length (mm)	Ultimate Load (kN)	Displacement (mm)	Failure Mode	Particular
C1	629	361	19.04	7.9	U-head fracture	-
C2	623	351	16.01	9.6	U-head fracture	epoxy resin was not used
C3	626	350	19.59	9.8	U-head fracture	-
Averaged 1	626	354	18.22	9.1	-	-
A4	627	355	14.94	8.8	U-head fracture	friction was increased
A5	629	349	12.95	10.2	U-head fracture	-
A6	623	343	11.20	9.9	U-head fracture	local failure occurs
A7	627	351	13.58	11.0	U-head fracture	-
A8	630	356	12.96	11.6	U-head fracture	-
A9	630	354	9.29	11.4	U-head fracture	local failure occurs
Averaged 2	628	351	12.49	10.5	-	-

**Table 3:** Comparison of the theoretical result and the tensile test result.

Type	Thin-Shell	Thick-Shell	Batch C	Batch A
The ultimate load (kN)	18.07	16.21	18.22	12.49

## Data Availability

Not applicable.

## References

[1] Feng P., Zhang P., Meng X., Ye L. (2014). Mechanical analysis of stress distribution in a carbon fiber-reinforced polymer rod bonding anchor. Polymers.

[2] Raza A., Rashedi A., Rafique U., Hossain N., Akinyemi B., Naveen J. (2021). On the structural performance of recycled aggregate concrete columns with glass fiber-reinforced composite bars and hoops. Polymers.

[3] Li Z., Chen C., Mi R., Gan W., Dai J., Jiao M., Xie H., Yao Y., Xiao S., Hu L. (2020). A strong, tough, and scalable structural material from fast-growing bamboo. Adv. Mater..

[4] Wang Y., Wang X., Li Y., Huang P., Yang B., Hu N., Fu S. (2021). High-performance bamboo steel derived from natural bamboo. ACS Appl. Mater. Inter..

[5] Javadian A., Wielopolski M., Smith I.F.C., Hebel D.E. (2016). Bond-behavior study of newly developed bamboo-composite reinforcement in concrete. Constr. Build. Mater..

[6] Agarwal A., Nanda B., Maity D. (2014). Experimental investigation on chemically treated bamboo reinforced concrete beams and columns. Constr. Build. Mater..

[7] Mali P.R., Datta D. (2020). Experimental evaluation of bamboo reinforced concrete beams. J. Build. Eng..

[8] Moroz J.G., Lissel S.L., Hagel M.D. (2014). Performance of bamboo reinforced concrete masonry shear walls. Constr. Build. Mater..

[9] Puri V., Chakrabortty P. Behavior of sustainable prefabricated bamboo reinforced wall panels under concentrated load. Proceedings of the International Conference on Sustainable Infrastructure 2017.

[10] Zhao W.F., Zhou J., Bu G.B. (2012). Application technology of bamboo reinforced concrete in building structure. Appl. Mech. Mater..

[11] Wang C., Liu Y., Zheng X., Wu J. (2019). Experimental investigation of a precast concrete connection with all-steel bamboo-shaped energy dissipaters. Eng. Struct..

[12] Xiao Y., Zhou Q., Shan B. (2010). Design and construction of modern bamboo bridges. J. Bridge Eng..

[13] Archila H., Kaminski S., Trujillo D., Zea Escamilla E., Harries K.A. (2018). Bamboo reinforced concrete: A critical review. Mater. Struct..

[14] Kathiravan N.S., Manojkumar R., Jayakumar P., Kumaraguru J., Jayanthi V. (2021). State of art of review on bamboo reinforced concrete. Mater. Today.

[15] Ghavami K. (2005). Bamboo as reinforcement in structural concrete elements. Cem. Concr. Compos..

[16] Mali P.R., Datta D. (2019). Experimental study on improving bamboo concrete bond strength. Adv. Concr. Constr..

[17] Umeonyiagu I.E., Nwobi-Okoye C.C. (2019). Modelling and multi objective optimization of bamboo reinforced concrete beams using ANN and genetic algorithms. Eur. J. Wood Wood Prod..

[18] Hu X. (1957). Water proofing treatment of bamboo bar (bamboo bar is used instead of steel bar in reinforced concrete). J. Tongji Univ..

[19] Kute S.Y., Wakchaure M.R. (2013). Performance evaluation for enhancement of some of the engineering properties of bamboo as reinforcement in concrete. J. Inst. Eng. Ser. A.

[20] Fang H., Wu Q., Hu Y., Wang Y., Yan X. (2013). Effects of thermal treatment on durability of short bamboo-fibers and its reinforced composites. Fiber. Polym..

[21] Lima H.C., Willrich F.L., Barbosa N.P., Rosa M.A., Cunha B.S. (2008). Durability analysis of bamboo as concrete reinforcement. Mater. Struct..

[22] Djamaluddin R., Yamaguchi K., Hino S. (2014). Mechanical behavior of the U-anchor of super-CFRP rod under tensile loading. J. Compos. Mater..

[23] Fan H., Vassilopoulos A.P., Keller T. (2017). Pull-out behavior of CFRP single-strap ground anchors. J. Compos. Constr..

[24] Li X. (2011). Test Research and Analysis on Mechanical Properties of Phyllostachys Pubescens. Master’s Thesis.

[25] Zeng Q., Li S., Bao X. (1992). Effect of bamboo nodal on mechanical properties of bamboo wood. For. Sci..

[26] Shao Z., Huang S., Wu F., Zhou L., Clement A. (2008). Study on the difference of structure and strength between internodes and nodes of moso bamboo. Bamboo Res. Trans..

